# Cell landscape atlas for patients with chronic thromboembolic pulmonary hypertension after pulmonary endarterectomy constructed using single-cell RNA sequencing

**DOI:** 10.18632/aging.203168

**Published:** 2021-06-21

**Authors:** Ran Miao, Xingbei Dong, Juanni Gong, Yidan Li, Xiaojuan Guo, Jianfeng Wang, Qiang Huang, Ying Wang, Jifeng Li, Suqiao Yang, Tuguang Kuang, Jun Wan, Min Liu, Zhenguo Zhai, Jiuchang Zhong, Yuanhua Yang

**Affiliations:** 1 Medical Research Center, Beijing Chao-Yang Hospital, Capital Medical University, Beijing 100020, China; 2 Key Laboratory of Respiratory and Pulmonary Circulation Disorders, Institute of Respiratory Medicine, Beijing 100020, China; 3 Chinese Academy of Medical Sciences and Peking Union Medical College, Beijing 100730, China; 4 Department of Respiratory and Critical Care Medicine, Beijing Chao-Yang Hospital, Capital Medical University, Beijing 100020, China; 5 Department of Echocardiography, Beijing Chao-Yang Hospital, Capital Medical University, Beijing 100020, China; 6 Department of Radiology, Beijing Chao-Yang Hospital, Capital Medical University, Beijing 100020, China; 7 Department of Interventional Radiology, Beijing Chao-Yang Hospital, Capital Medical University, Beijing 100020, China; 8 Department of Pathology, Beijing Chao-Yang Hospital, Capital Medical University, Beijing 100020, China; 9 Department of Pulmonary and Critical Care Medicine, China-Japan Friendship Hospital, Beijing 100029, China; 10 Department of Radiology, China-Japan Friendship Hospital, Beijing 100029, China; 11 Heart Center and Beijing Key Laboratory of Hypertension, Beijing Chao-Yang Hospital, Capital Medical University, Beijing 100020, China

**Keywords:** chronic thromboembolic pulmonary hypertension, single-cell RNA sequencing, gene ontology enrichment analysis

## Abstract

This study aimed to construct an atlas of the cell landscape and comprehensively characterize the cellular repertoire of the pulmonary endarterectomized tissues of patients with chronic thromboembolic pulmonary hypertension (CTEPH). Five pulmonary endarterectomized tissues were collected. 10× Genomics single-cell RNA sequencing was performed, followed by the identification of cluster marker genes and cell types. Gene Ontology (GO) enrichment analysis was conducted. Seventeen cell clusters were characterized, corresponding to 10,518 marker genes, and then classified into eight cell types, including fibroblast/smooth muscle cell, endothelial cell, T cell/NK cell, macrophage, mast cell, cysteine rich secretory protein LCCL domain containing 2 (CRISPLD2)+ cell, cancer stem cell, and undefined. The specific marker genes of fibroblast/smooth muscle cell, endothelial cell, T cell/NK cell, macrophage, mast cell, and cancer stem cell were significantly enriched for multiple functions associated with muscle cell migration, endothelial cell migration, T cell activation, neutrophil activation, erythrocyte homeostasis, and tissue remodeling, respectively. No functions were significantly enriched for the marker gene of CRISPLD2+ cell. Our study, for the first time, provides an atlas of the cell landscape of the pulmonary endarterectomized tissues of CTEPH patients at single-cell resolution, which may serve as a valuable resource for further elucidation of disease pathophysiology.

## INTRODUCTION

Chronic thromboembolic pulmonary hypertension (CTEPH) is a rare and debilitating disorder characterized by pulmonary artery obstruction with unresolved, organized thromboemboli [1, 2]. Due to delayed symptoms or misdiagnosis, CTEPH is typically diagnosed at an advanced stage, and as a result, disease prognosis is poor, with a < 40% 5-year survival rate [3, 4]. The only curative treatment currently available is pulmonary endarterectomy (PEA) for improving quality of life and prolonging survival [5–7]. However, not all patients are eligible for surgery, and alternative treatments are still required. Moreover, the exact pathophysiology of CTEPH remains largely unknown. To improve clinical outcomes, improved understanding of the detailed mechanisms involved in CTEPH is crucial.

The occurrence and development of CTEPH are complex processes involving various genes, multiple cell types, and a large number of signal transduction systems [8]. In particular, the roles of multiple cell types in disease development are gaining increasing attention. It has been reported that accumulation of circulating inflammatory cells [9] and excessive proliferation and migration of smooth muscle and endothelial cells [10–12] are involved in pulmonary arterial remodeling during CTEPH. Moreover, crosstalk between endothelial-like and myofibroblast-like cells has been shown to play a key role in endothelial cell dysfunction, contributing to vascular lesion in CTEPH [13]. Despite these findings, the cell types contributing to CTEPH progression have not been fully elucidated. A clear and complete atlas of the cellular repertoire that participates in the occurrence and development of CTEPH is vital for the development of effective diagnostic and therapeutic strategies to prevent CTEPH and decrease mortality.

The recent development of single-cell RNA sequencing (scRNA-seq) technology has provided new perspectives on the complex biological systems and key functions associated with disease development [14–16]. In the present study, we applied scRNA-seq to comprehensively characterize the cell cluster composition in the pulmonary endarterectomized tissues of patients with CTEPH for the first time. Our results revealed that the cell populations in pulmonary endarterectomized tissues include fibroblast/smooth muscle cell, T cell/natural killer (NK) cell, macrophage, cysteine rich secretory protein LCCL domain containing 2 (CRISPLD2)+ cell, cancer stem cell, mast cell, endothelial cell, and undefined. This study, for the first time, provided a comprehensive and unique resource revealing CTEPH cellular typing at single-cell resolution. Uncovering the function of these cell populations will help us to better understand CTEPH pathophysiology.

## RESULTS

### Quality control analysis

**Figure 1:**
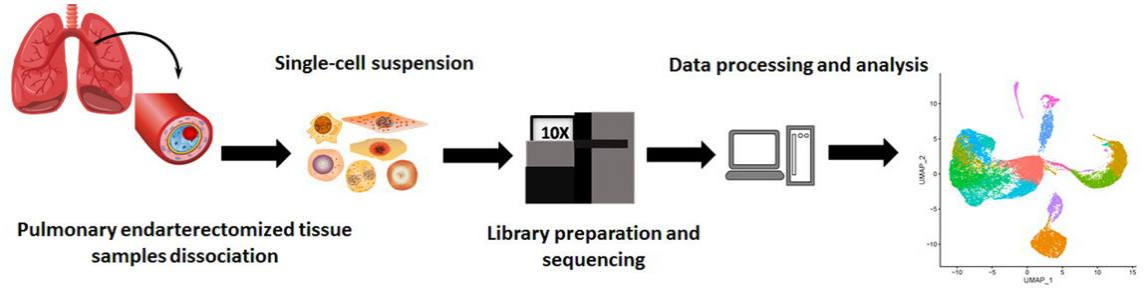
Schematic of the experimental design for single-cell RNA sequencing.

To construct an atlas of the cell repertoire in CTEPH, we generated scRNA-seq profiles of pulmonary endarterectomized tissues from five CTEPH patients undergoing PEA. Tissue samples were digested into single-cell suspensions and analyzed by droplet-based single-cell transcriptome profiling using the 10× Genomics Single Cell Gene Expression Chromium system (https://www.10xgenomics.com/products/single-cell-gene-expression) (Figure 1). DNA sequencing reads were mapped to the hg19 human reference genome. We generated five-feature barcode matrices for each of the five samples independently, and these matrices were then aggregated. Subsequently, we filtered and collected 27,140 cells. By visualizing histograms representing gene number and unique molecular identifier (UMI) count, individual cells and their gene expression in the sample could be evaluated. For species for which mitochondrial genome information is available, the percentage of mitochondrial genes in cells was calculated to filter cells with relatively high mitochondrial gene content. Gene numbers, UMI counts, and percentage of mitochondrial genes are represented using violin plots (Supplementary Figure 1).

### Overall characteristics of cell cluster composition in pulmonary tissues after endarterectomy of CTEPH patients

**Figure 2:**
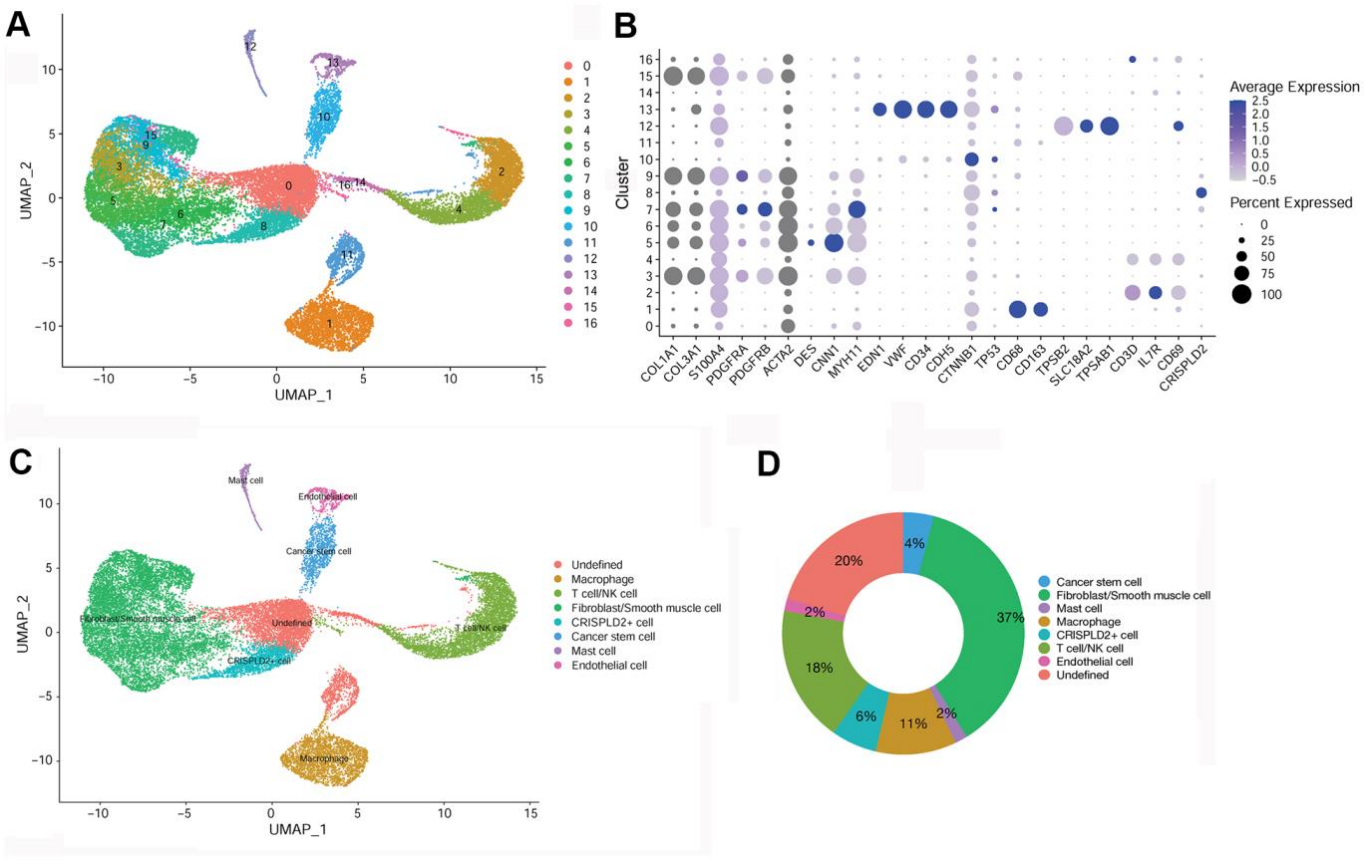
**Identification of key marker genes and overall characteristics of cell cluster compositions in pulmonary endarterectomized tissues of CTEPH patients.** (**A**) UMAP plot of 17 clusters. (**B**) Bubble diagram showing the key marker genes in 17 clusters. (**C**) UMAP plot of eight cell types. Based on cell markers, clusters 3, 5, 6, 7, 9, and 15 were annotated as fibroblasts/smooth muscle cells. Clusters 2 and 4 were classified as T-/NK cells. Clusters 1, 8, 10, 12, and 13 were classified as macrophages and CRISPLD2+, cancer stem, mast, and endothelial cells, respectively. Clusters 0, 11, 14, and 16 were classified as undefined. (**D**) Pie chart showing the proportion of each cell type among total cells. Abbreviations: CTEPH: chronic thromboembolic pulmonary hypertension; UMAP: Uniform Manifold Approximation and Projection; CRISPLD2: cysteine-rich secretory protein LCCL domain containing 2.

After quality filtering and normalization of gene expression, dimension reduction involving the top 20 components was conducted. As a result, a total of 17 cell clusters (clusters 0–16) was characterized (Figure 2A), corresponding to 10,518 marker genes. We then determined marker genes that were uniquely expressed in each cell cluster in comparison with all other clusters. The key marker genes for each cell cluster are presented in Figure 2B. Based on cell markers, fibroblast/smooth muscle cells were composed of clusters 3, 5, 6, 7, 9, and 15, and accounted for the largest proportion (37%) of total cells. Clusters 2 and 4 were classified as T-/NK cells, with a relative percentage of 18%. Clusters 1, 8, 10, 12, and 13 were classified as macrophages (11%), CRISPLD2+ cells (6%), cancer stem cells (4%), mast cells (2%), and endothelial cells (2%), respectively. Clusters 0, 11, 14, 16 were categorized as ‘undefined’ (20%) (Figure 2C, 2D).

### Characterization of fibroblast/smooth muscle cell and endothelial cell in the pulmonary endarterectomized tissues of CTEPH patients

**Figure 3:**
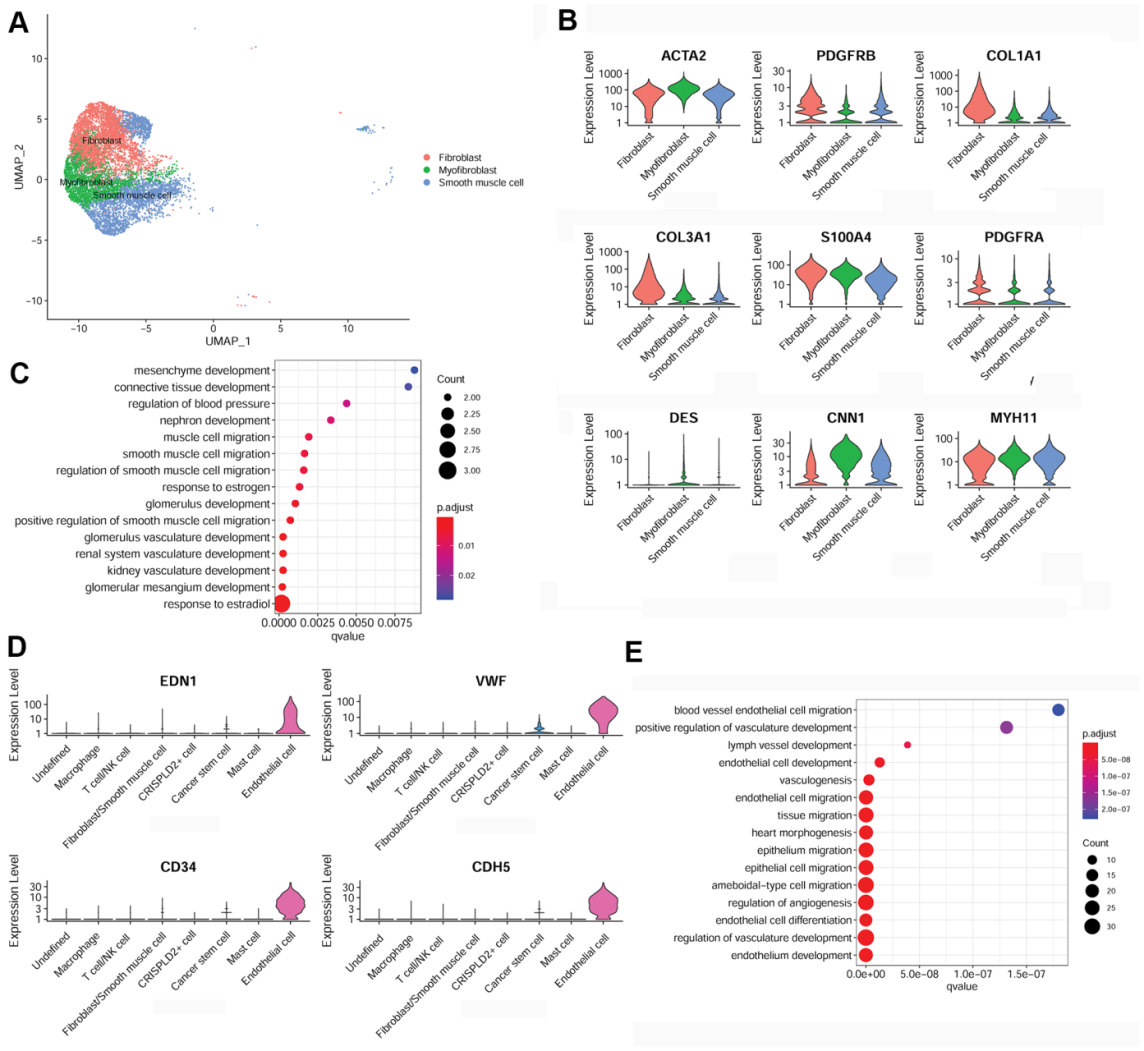
**Characterization of fibroblasts/smooth muscle cells and endothelial cells in the pulmonary endarterectomized tissues of CTEPH patients.** (**A**) UMAP plot of three subsets of fibroblasts/smooth muscle cells. (**B**) Violin plot illustrating fibroblast/smooth muscle cell/myofibroblast marker genes, including *ACTA2*, *PDGFRB*, *COL1A1*, *COL3A1*, *S100A4*, *PDGFRA*, *DES*, *CNN1*, and *MYH11.* (**C**) GO enrichment analysis of fibroblast/smooth muscle cell/myofibroblast markers. (**D**) Violin plot showing marker genes of endothelial cells, including *END1*, *VWF*, *CD34*, and *CDH5*. (**E**) GO enrichment analysis of endothelial cell marker genes. Abbreviations: CTEPH: chronic thromboembolic pulmonary hypertension; UMAP: Uniform Manifold Approximation and Projection; GO: Gene ontology.

To further examine fibroblast/smooth muscle cell clusters in CTEPH, Uniform Manifold Approximation and Projection (UMAP) were used to sub-cluster fibroblast/smooth muscle cells into three distinct subsets: fibroblasts, myofibroblasts, and smooth muscle cells (Figure 3A). Specific marker genes for the fibroblast/smooth muscle cell cluster were identified, including smooth muscle actin alpha 2 (*ACTA2*), platelet-derived growth factor receptor beta (*PDGFRB*), collagen type I alpha 1 chain (*COL1A1*), collagen type III alpha 1 chain (*COL3A1*), S100 calcium binding protein A4 (*S100A4*), platelet-derived growth factor receptor alpha (*PDGFRA*), desmin (*DES*), calponin 1 (*CNN1*), and myosin heavy chain 11 (*MYH11*) (Figure 3B). Moreover, these marker genes were significantly enriched for multiple functions associated with smooth muscle cell migration (Figure 3C).

In addition, specific marker genes of endothelial cells were determined to be endothelin 1 (*END1*), von Willebrand factor (*VWF*), *CD34*, and cadherin 5 (*CDH5*) (Figure 3D). These were mainly enriched for functions associated with cell migration, such as endothelial cell migration, epithelium migration, and ameboid-type cell migration (Figure 3E).

### Characterization of T cell/NK cell, macrophage, and mast cell in the pulmonary endarterectomized tissues of CTEPH patients

**Figure 4:**
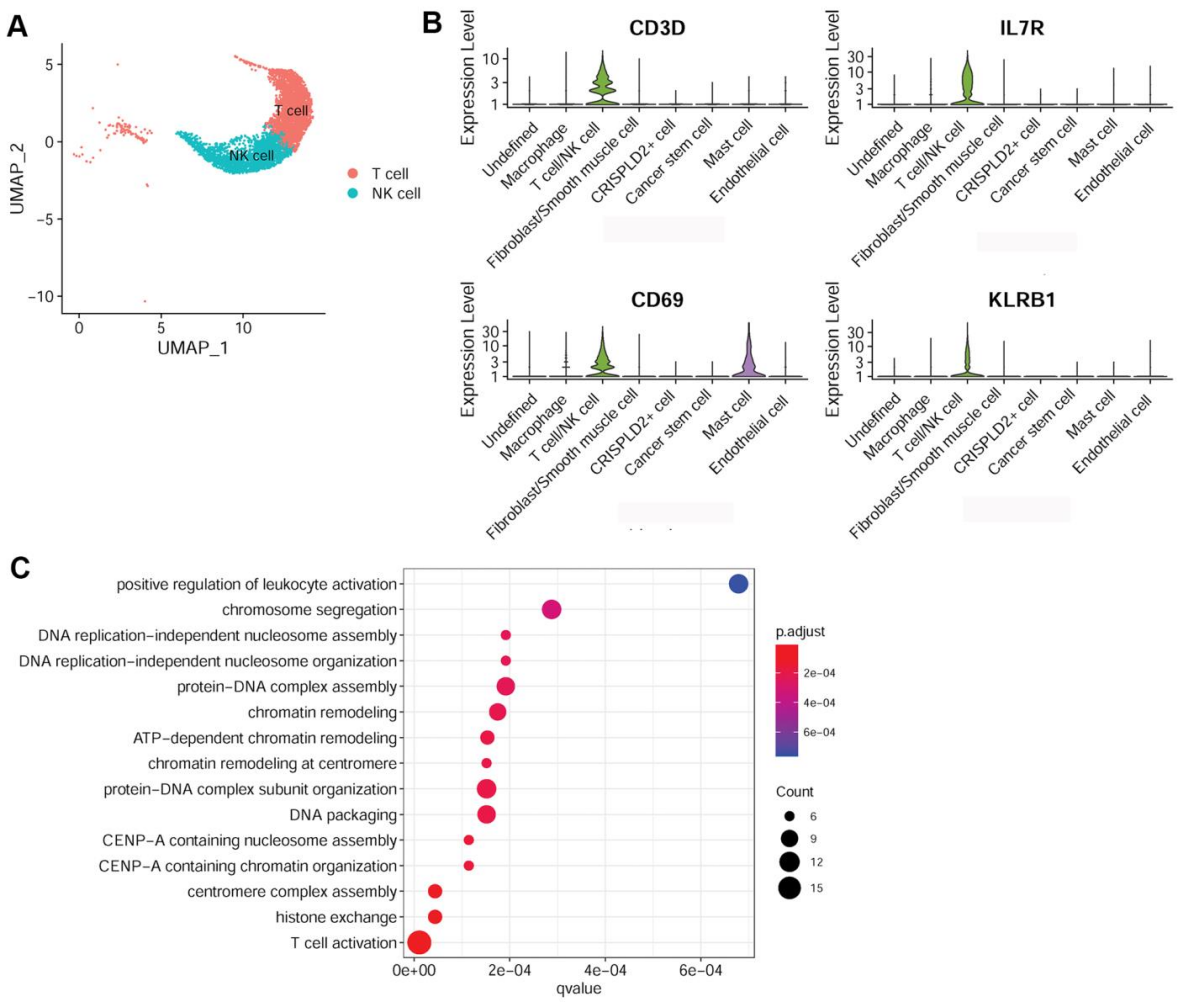
**Characterization of T-/NK cells in pulmonary endarterectomized tissues of CTEPH patients.** (**A**) UMAP plot of T and NK cells. (**B**) Violin plot showing the marker genes of T cell/NK cells, including *CD3D*, *IL7R*, *CD69*, and *KLRB1.* (**C**) GO enrichment analysis of marker genes of T-/NK cells. Abbreviations: CTEPH: chronic thromboembolic pulmonary hypertension; UMAP: Uniform Manifold Approximation and Projection; GO: Gene ontology.

**Figure 5:**
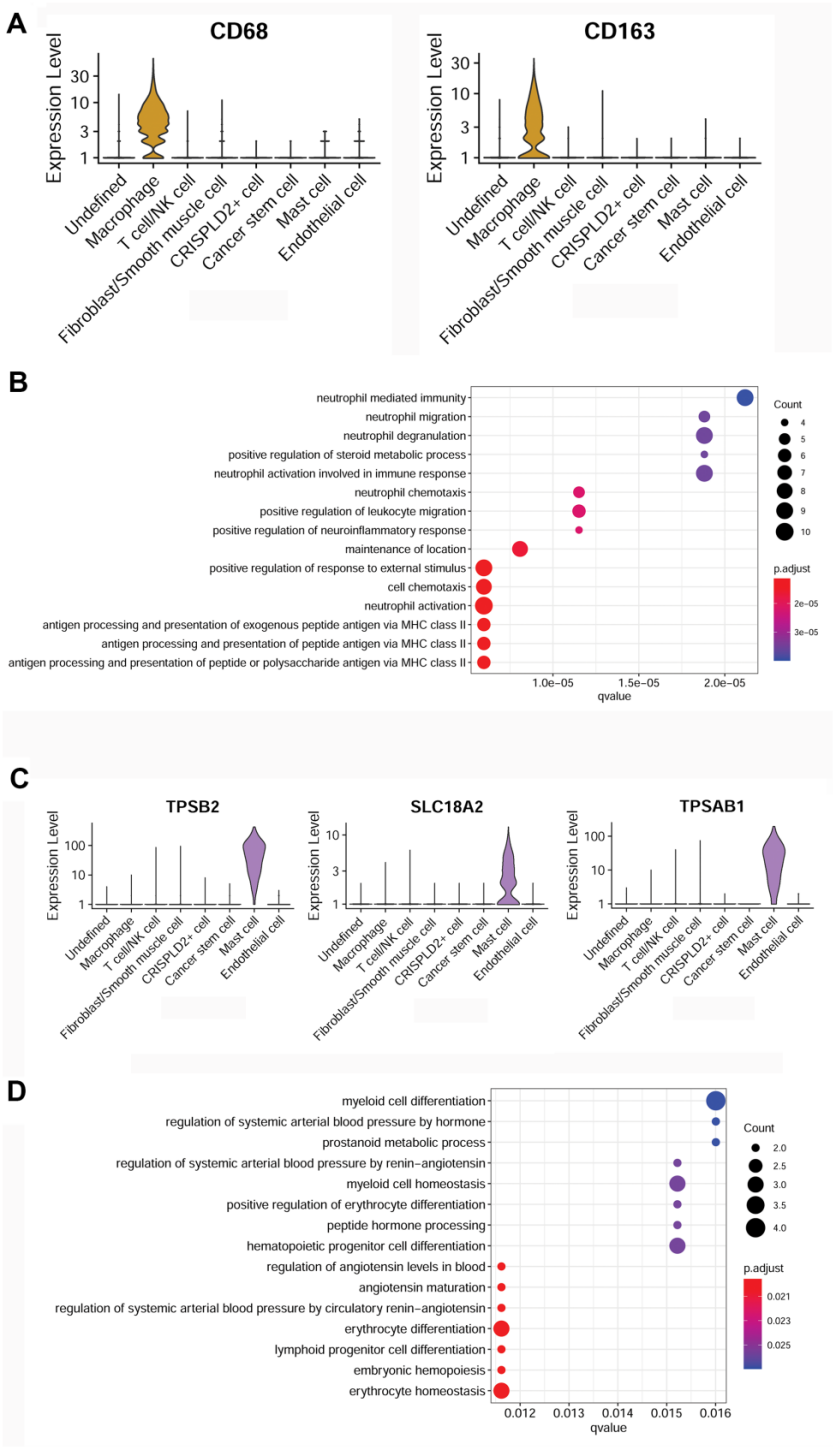
**Characterization of macrophages and mast cells in pulmonary endarterectomized tissues of CTEPH patients.** (**A**) Violin plot showing the marker genes of macrophages, including *CD68* and *CD163.* (**B**) GO enrichment analysis of marker genes of macrophages. (**C**) Violin plot showing mast cell marker genes, including *TPSB2*, *SLC18A2*, and *TPSAB1.* (**D**) GO enrichment analysis of mast cell marker genes. Abbreviations: CTEPH: chronic thromboembolic pulmonary hypertension; GO: Gene ontology.

To better understand the immune mechanisms underlying CTEPH, we further characterized T-/NK cells, macrophages, and mast cells in pulmonary endarterectomized tissues of CTEPH patients. The T-/NK cell cluster was also subdivided into T- and NK cells, as shown in the UMAP plot (Figure 4A). Figure 4B shows violin plots of the specific marker genes of the T-/NK cell cluster, including *CD3D*, interleukin 7 receptor (*IL7R*), *CD69*, and killer cell lectin-like receptor B1 (*KLRB1*). Further Gene Ontology (GO) enrichment analysis showed that T cell activation was an important function significantly enriched in the marker genes identified in T-/NK cells (Figure 4C). Specific macrophage marker genes were identified as *CD68* and *CD163* (Figure 5A), which were significantly involved in key functions in neutrophil activation, cell chemotaxis, and antigen processing and presentation via MHC Class II molecules (Figure 5B). In addition, the specific marker genes of mast cells were revealed to be tryptase beta 2 (*TPSB2*), solute carrier family 18 member A2 (*SLC18A2*), and tryptase alpha/beta 1 (*TPSAB1*) (Figure 5C). The significantly enriched GO functions of these mast cell marker genes included erythrocyte differentiation and homeostasis (Figure 5D).

### Characterization of other cell clusters in pulmonary endarterectomized tissues of CTEPH patients

**Figure 6:**
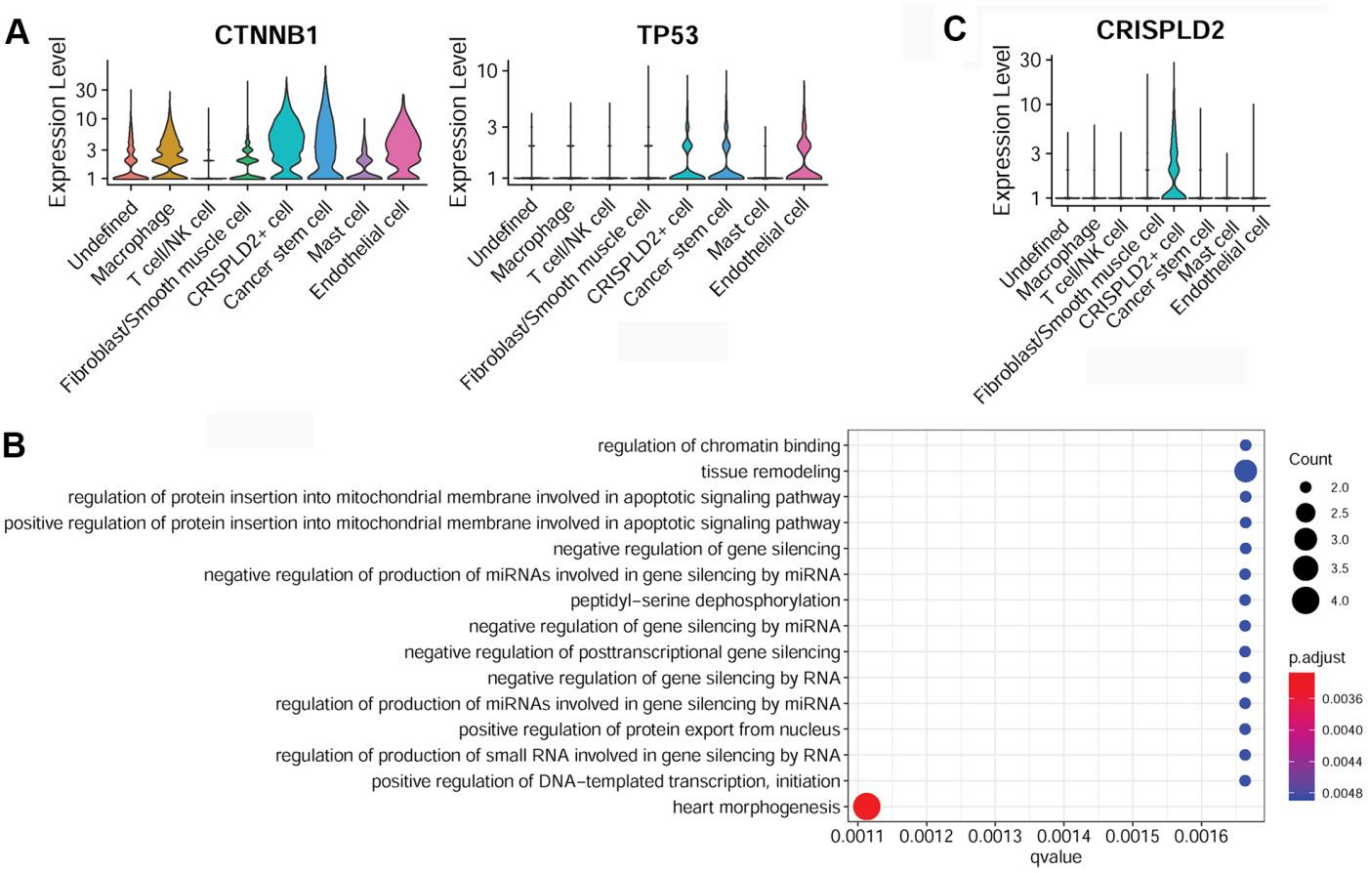
**Characterization of cancer stem cells and CRISPLD2+ cells in pulmonary endarterectomized tissues of CTEPH patients.** (**A**) Violin plot illustrating cancer stem cell marker genes, including *CTNNB1* and *TP53.* (**B**) GO enrichment analysis of cancer stem cell marker genes. (**C**) Violin plot showing the *CRISPLD2* marker of CRISPLD2+ cells. Abbreviations: CTEPH: chronic thromboembolic pulmonary hypertension; CRISPLD2: cysteine-rich secretory protein LCCL domain containing 2; GO: Gene ontology.

Specific marker genes of cancer stem cells were determined to be catenin beta 1 (*CTNNB1*) and tumor protein P53 (*TP53*) (Figure 6A). The over-represented GO function in cancer stem cell marker genes was categorized as ‘tissue remodeling’ (Figure 6B). The specific marker gene of CRISPLD2+ cells was identified as *CRISPLD2* (Figure 6C). However, no significant GO function was enriched for this gene.

## DISCUSSION

CTEPH is a potentially curable disease, but its pathophysiology is not fully understood. There is limited research about the cells that make up the endarterectomized tissues. The present study, for the first time, investigated the cell landscape of pulmonary endarterectomized tissues of CTEPH patients using scRNA-seq. We found 17 cell clusters that could be divided into eight cell populations based on marker genes, as follows: including fibroblast/smooth muscle cell, T cell/NK cell, macrophage, CRISPLD2+ cell, cancer stem cell, mast cell, endothelial cell, and undefined. The functions of these cell populations with respect to CTEPH and their marker genes merit further discussion.

CTEPH is characterized by pulmonary vascular remodeling, in which fibroblasts, smooth muscle and endothelial cells have been shown to play important roles [17]. Vascular remodeling typically manifests as medial hypertrophy caused by increasing cell proliferation, or by attenuating apoptosis of vascular smooth muscle cells, as well as lumen obliteration, resulting from the over-proliferation of endothelial cells [17]. Moreover, lung fibroblasts have been reported to participate in developmental airway remodeling and tissue repair after inflammatory injury involving small airways [18]. In addition, pulmonary endothelial cells have been shown to be key regulators of pulmonary artery smooth muscle cell behavior in CTEPH [19]. In our study, fibroblast/smooth muscle cells/myofibroblasts accounted for the largest proportion (37%) of total cells, and endothelial cells accounted for only 2%. These data suggested an essential role for fibroblasts/smooth muscle cells/myofibroblasts and endothelial cell populations in CTEPH. Furthermore, specific marker genes of fibroblast/smooth muscle cells were identified, such as *PDGFRB*, *COL1A1*, and *COL3A1*, which were significantly enriched for multiple functions associated with smooth muscle cell migration. It has been reported that CTEPH endothelial cells can exhibit pro-fibrotic and pro-inflammatory phenotypes, as demonstrated by the expression of genes (*COL1A1* and *COL3A1*) involved in extracellular matrix production and fibril organization [20]. In addition, PDGF is a potent mitogen for cells of mesenchymal origin, such as fibroblasts and smooth muscle cells [21]. Previous studies have revealed that smooth muscle 22 (SM22) and α-smooth muscle actin (α-SMA) are major marker genes of smooth muscle cells [22]. The PDGFRB pathway can inhibit the expression of SM22 and α-SMA and thus play crucial roles in phenotypic modulation of smooth muscle cells, facilitating the infiltration of inflammatory cells [23]. *PDGFRB* has been also identified to play important roles in CTEPH development [24]. Specific endothelial cell marker genes are *END1* and *CDH5*, and these are mainly enriched for functions associated with endothelial cell migration. PKC-mediated *END1* expression in endothelial cells has been shown to promote macrophage activation in atherogenesis [25]. A recent study has shown that *END1* expression by endothelial cells may contribute to thrombofibrosis in the development of CTEPH [26]. *CDH*5, an endothelial marker gene, has been shown to promote endothelial–mesenchymal transition [27]. Sakao et al. showed that *CDH5* is implicated in endothelial cell activation in CTEPH [13]. Based on our results, we speculated that fibroblast/smooth muscle cell/myofibroblast and endothelial cell populations may be involved in CTEPH development via the regulation of important functions associated with smooth muscle or endothelial cell migration.

Accumulating evidence has revealed the crucial role of dysregulated immune responses in CTEPH development [28], and such immune responses relate to either innate or adaptive immunity. Macrophages, NK cells, and mast cells participate in innate immunity, while T and B cells are implicated in adaptive immunity [28]. In CTEPH patients, macrophages and activated T and B lymphocytes are involved in thrombotic and atherosclerotic lesions [29]. Macrophages are first-line myeloid leucocytes in pulmonary lesions in patients with pulmonary hypertension [30]. Mast cells are tissue-resident immune cells that have key functions in the immune system through the production of inflammatory cytokines. Macrophages and mast cells play a pathological role in CTEPH via production of inflammatory cytokines, recruitment of other immune cells, and local inflammation and damage [28]. Moreover, *CD68* and *CD163* are two macrophage-associated markers, whose expression levels are associated with adverse outcomes in human atherosclerosis [31]. CD68- and CD163-positive tumor-associated macrophages are reported as key factors in tumor growth and metastasis through releasing chemokines such as inflammatory growth factors [32]. Our results showed that specific macrophage marker genes (such as *CD68* and *CD163*) are significantly involved in key functions related to neutrophil activation, cellular chemotaxis, and antigen processing and presentation via MHC Class II molecules. Neutrophils are early responders and are recruited to respond to chemokines produced by tissue-resident immune cells such as macrophages and mast cells. In addition, it has been reported that CD3+ T cells accumulate in atherosclerotic and thrombotic lesions of CTEPH patients [29]. CD69 has been shown to be involved in immune cell homeostasis, which can activate T cell-mediated immune responses via regulating Th17 cell differentiation [33]. CD69 has also been identified as being inhibitory to cardiac inflammation and heart failure progression in autoimmune myocarditis [34]. Our analysis showed that specific T-/NK cell marker genes (such as *CD3D* and *CD69*) were remarkably enriched for T cell activation. It can be speculated that during CTEPH, the accumulation of macrophages and mast cells promotes the production of inflammatory cytokines and chemokines and subsequently induces T cell activation.

Notably, *CRISPLD2*, which encodes a secreted protein, has been shown to increase the anti-inflammatory effects of glucocorticoids to mediate immune responses in airway smooth muscle cells [35]. *CRISPLD2* also functions as an endogenous anti-inflammatory gene in lung fibroblasts, and is an inhibitor of pro-inflammatory signaling in lung epithelial cells [36]. Based on our results, we speculated that CRISPLD2+ cells may be involved in CTEPH via the regulation of anti-inflammatory processes and subsequent immune responses. In addition to these, cancer stem cell clusters were also identified as a relatively newly recognized population in pulmonary endarterectomized tissues in CTEPH patients. Specific cancer stem cell marker genes are *CTNNB1* and *TP53*. *CTNNB1* expression is elevated in lung tissues from patients with idiopathic pulmonary arterial hypertension and CTNNB1 is involved in vascular remodeling via the Wnt/β-catenin pathway [37]. It has been shown that, in pulmonary artery smooth muscle cells, inhibition of *TP53* results in upregulation of glycolysis and downregulation of mitochondrial respiration, indicating a proliferative phenotype resembling that of cancer cells [38]. Feng et al. demonstrated that miR-31a-5p targets *TP53* to promote primary hypertension through accelerating proliferation and inhibiting apoptosis of arterial smooth muscle cells, suggesting a potential role of *TP53* in pulmonary arterial hypertension [39]. In our study, specific cancer stem cell marker genes were enriched for tissue remodeling functions. We hypothesized that cancer stem cells may be involved in CTEPH by participating in tissue remodeling.

However, one of the limitations of our study was the relatively small sample size, which might be a consequence of the single-center study as here, the short duration of patient inclusion, and the low incidence of disease. A multicenter research involving a larger sample size may, in the future, provide additional strong evidence to support our findings reported here. Moreover, unidentified cells accounted for 20% of all cells. Elucidation of the fate of this large fraction of cells may provide new perspectives to better reveal the pathological mechanisms involved in CTEPH. Lastly, this study, for the first time, conducted scRNA-seq to reveal the cellular composition involved in CTEPH—essentially at single-cell resolution—and represents an exploratory study that provides a valuable resource facilitating researchers in future investigations of the possible mechanisms underlying CTEPH. However, CTEPH patients are rare and there are fewer CTEPH patients who eligible to receive surgical interventions. Coupled with the fact that this was a single-center study, it is difficult to assemble sufficient patient numbers and samples to carry out subsequent experiments in a short period of time. Therefore, we did not examine the pathology of cell types in CTEPH patients via histological analysis by immunohistochemistry to confirm our findings obtained from scRNA-seq, nor did we perform functional examinations of these cells. Further studies are still needed to combine clinical characterization of patients with experimental validation of existing clusters to explore possible mechanisms underpinning the disease.

In conclusion, our scRNA-seq analysis revealed that fibroblast/smooth muscle cells/myofibroblasts and endothelial cell populations may be involved in CTEPH development by participating in important functions associated with muscle or endothelial cell migration involving pulmonary vascular remodeling. Macrophage, mast cell, T cell/NK cell, and CRISPLD2+ cell are implicated in CTEPH via their involvement in inflammatory and immune responses. Cancer stem cells may be involved in CTEPH via participation in tissue remodeling. Collectively, our study provided the first atlas detailing the cellular landscape in pulmonary endarterectomized tissues of CTEPH patients, which will help us to better understand the pathological mechanisms involved in this disease.

## MATERIALS AND METHODS

Requirements for ethical approval and written informed consent were waived for this study because discarded pulmonary endarterectomized tissues of CTEPH patients were used here.

### Handling of pulmonary endarterectomized tissue samples and dissociation to a single-cell suspension

Five consecutive patients were enrolled from October 2019 to June 2020 and definitively diagnosed with CTEPH. Pulmonary endarterectomized tissues from five CTEPH patients undergoing PEA were collected and stored in a tissue preservation solution. The baseline characteristics of these CTEPH patients are shown in Table 1.

**Table 1:** The baseline characteristics of CTEPH patients used for single-cell RNA sequencing.

**Features**	**CTEPH patients (N = 5)**
Female/male	1/4
Age, years	45.00 ± 13.34
BMI, (kg/m^2^)	25.18 ± 1.18
mPAP, mmHg	54.40±3.21
WHO FC (I-II; III-IV)	1/4
CI, L/(min·m^2^)	1.78±0.13
Family history of blood clots	No
Smoking	2
Long period of inactivity	No
Other CTEPH risk factors	
Pulmonary embolism	3
Venous thromboembolism	2

Abbreviations: CTEPH: chronic thromboembolic pulmonary hypertension; BMI: body mass index; mPAP: mean pulmonary arterial pressure; WHO FC: World Health Organization function classification; CI: cardiac index.

The tissue samples were treated with enzymatic hydrolysate 1 at 30–40° C. Enzymatic hydrolysate 1 was prepared by adding a final concentration of 2.5 mg/mL collagenase I and 2.5 mM CaCl_2_ to RPMI 1640 cell culture medium. Then, 50 U/mL DNAseI was added to the enzymatic hydrolysate 1 to fully digest the tissue samples. After filtering, the samples were further digested using enzymatic hydrolysate 2, which was prepared by adding a final concentration of 1 mg/mL collagenase I, 1 mg/mL collagenase II, 0.2 mg/mL collagenase XI, 1.8 U/mL elastase, 50 U/mL DNAseI and 2.5 mM CaCl_2_ to RPMI 1640 medium. Digested tissue was macerated by passage through a 100 μM cell strainer, and red blood cell lysis buffer (Life Technologies, Ghent, Belgium) was added to eliminate potential interference from red blood cells. After terminating the reaction, cell pellets were collected by centrifugation. The cells were resuspended with deactivated magnetic beads, incubated at room temperature, added to the rinsed magnetic beads, and then collected by centrifugation. The cells were then resuspended again for preparation of single-cell suspensions.

### scRNA-seq

The single-cell suspensions were loaded onto a Chromium™ Single-Cell Instrument (10× Genomics, Pleasanton, CA, USA) to create barcoded single-cell gel beads in emulsion (GEM), followed by reverse transcription and cDNA amplification. scRNA-seq libraries were then generated. Briefly, cDNA was sheared to 200–300 bp and then subjected to end repair and A-tailing, adapter ligation and sample index labeling for polymerase chain reaction, and sample cleanup. After quality checks, scRNA-seq was performed on the Illumina NovaSeq platform to obtain DNA sequencing data.

### Data processing

All scRNA-seq data for the five samples were processed and quantified using Cell Ranger Software Suite v.3.1.0 software tools (https://support.10xgenomics.com). First, the sequenced reads were mapped to the hg19 human reference genome under the Cell Ranger ‘count’ module. We generated five-feature barcode matrices for each of the five samples independently. These matrices were then aggregated using the Cell Ranger ‘aggr’ module for subsequent analyses. Second, cells with gene numbers < 200 or > 7,500, mitochondrial gene expression ratio > 0.2, and at least one gene expressed in three cells were filtered using Seurat v.3.0 (https://satijalab.org/seurat/) [40]. Data were then normalized using the NormalizeData function (pbmc, normalization.method = "LogNormalize," scale.factor = 10000). The UMI count matrix was then converted to Seurat objects using Seurat v.3.0.

### Identification of cluster marker genes and cell types

After data normalization and scaling, and dimensionality reduction of the top-20 components, cell clusters were identified using the ‘FindClusters’ function in Seurat v.3.0 and then visualized using the UMAP method after running ‘RunUMAP.’ Marker genes corresponding to each cell cluster were identified based on differential analysis using the ‘FindMakers’ function of the Seurat package and Wilcoxon rank-sum tests. The differential expression thresholds were set as log-fold change > 1 and P value < 0.05. The marker genes were then used to annotate cell types using the SCSA tool [41]. Violin plots of key marker genes were constructed using ggplot2 [42].

### GO enrichment analysis

GO [43] enrichment analysis is a common approach for functional studies of large-scale genomic or transcriptomic data. GO enrichment analysis for all the marker genes in each cluster was conducted using clusterProfiler (3.14.3) [44].

## Supplementary Material

Supplementary Figure 1
