# Supplementary material for: Cell landscape atlas for patients with chronic thromboembolic pulmonary hypertension after pulmonary endarterectomy constructed using single-cell RNA sequencing
Source: Aging (Albany NY). 2021 Jun 21;13(12):16485–99. doi: 10.18632/aging.203168 (PMC8266372; doi:10.18632/aging.203168)
Supplement: Supplementary Figure 1 [file aging-13-203168-s001.pdf]

SUPPLEMENTARY FIGURE

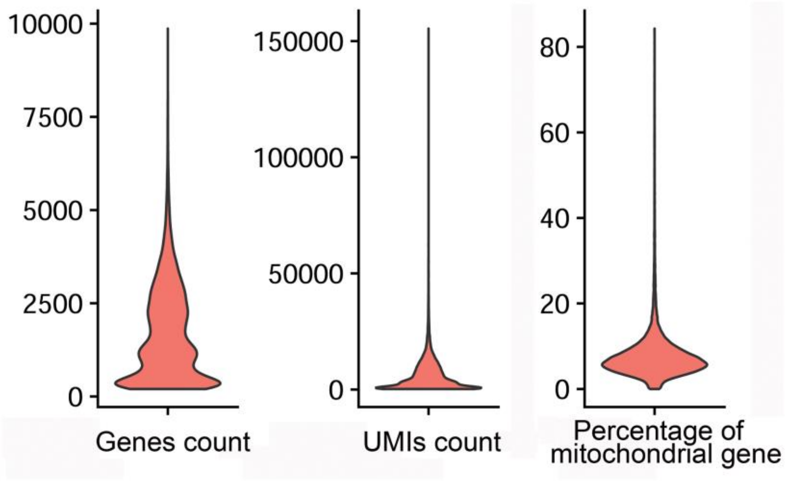

Supplementary Figure 1. Distribution of the number of genes (left), UMI counts (middle), and percentage of mitochondrial genes (right) in cells. UMI: unique molecular identifier.
